# Evidence of an intracellular creatine-sensing mechanism that modulates creatine biosynthesis via AGAT expression in human HAP1 cells

**DOI:** 10.1038/s41598-023-49860-1

**Published:** 2023-12-16

**Authors:** Michael B. Tropak, Ilona Tkachyova, Ray Gu, Alex Lee, Andreas Schulze

**Affiliations:** 1 Genetics and Genome Biology, The Hospital for Sick Children, Toronto, Canada; 2 Department of Pediatrics, University of Toronto, Toronto, Canada; 3 Department of Biochemistry, University of Toronto, Toronto, Canada

**Keywords:** Biochemistry, Biological techniques, Cell biology, Physiology, Medical research, Molecular medicine

## Abstract

Cellular homeostasis of creatine (CT), integral part of the energy buffering and transducing system connecting intracellular sites of ATP production and utilization, comprises of mechanisms that increase CT, i.e., biosynthesis and cellular uptake, and CT-lowering processes, such as export and non-enzymatic conversion to creatinine. The biosynthesis of CT is controlled by negative feedback loop via suppression of the rate-limiting enzyme arginine:glycine amidinotransferase (AGAT). Although the regulatory mechanism involved is not well understood, AGAT suppression is successfully used in patients with guanidinoacetate methyltransferase (GAMT) deficiency to reduce the neurotoxic accumulation of the AGAT-mediated guanidinoacetate production by supplementing patients with CT. Utilizing the CT-dependent feedback loop for the upregulation of AGAT expression may well represent a therapeutic target for an additional CT deficiency syndrome, the CT transporter (CrT) defect, for which no effective treatment option is available so far. We have used CRISPR to tag the C-terminus of AGAT with a nanoluc luciferase (NLuc) reporter in HAP1 cells. A biphasic decay of AGAT-NLuc in response to increasing extracellular CT was observed, whereas the decrease in AGAT-NLuc expression was directly proportional to the rise in intracellular CT levels with an approximate IC50 of 1–2 mM. CRISPR generated HAP1 CrT null cells and HAP1 CrT null cells stably expressing a CrT-GFP fusion protein further demonstrated that the biphasic response to extracellular CT is mediated by a high-affinity (Km 9–10 µM) CrT dependent, saturable mechanism and a CrT independent, unsaturable uptake process. The direct response to intracellular CT suggests the existence of an intracellular CT sensing system enabling a dynamic cell response to changing CT concentration that is relevant for cellular CT homeostasis.

## Introduction

Creatine (CT) and phosphocreatine serve as cytosolic energy transducers to connect the intracellular sites of ATP production and utilization^1,2^. Intracellular CT levels are determined by the rate of CT synthesis and degradation as well as import into and export from the cell. The synthetic pathway involves the rate limiting enzyme L-Arginine: glycine amidinotransferase (AGAT, gene: *GATM*) which produces guanidinoacetate (GAA) by transfer of the guanidino group from arginine (Arg) to glycine. *S*-Adenosyl-L-methionine: N-guanidinoacetate methyltransferase (GAMT, gene: *GAMT*) ads a methyl group onto GAA forming CT. The high affinity Na^+^/Cl^-^ dependent CT transporter (CrT, gene: *SLC6A8*) imports extracellular CT into the cell against a large concentration gradient, e.g. from 20–50 µM in plasma^3^ to ~ 8 mmol/kg in brain, ~ 2 mmol/kg in liver, 3 mmol/kg in kidney^4^, and 20–50 mmol/kg in striated muscle^5,6^. There is also evidence for a CrT-independent, low-affinity process of CT uptake that is linear and not saturable^7,8^. CT is non-enzymatically degraded to creatinine at a constant rate. The monocarboxylate transporter 12 (MCT12, gene: *SLC16A12*) facilitates the export of CT from sinusoidal hepatocytes^9^. MCT12 is also highly expressed in the kidney and retina^10^. How intracellular CT homeostasis is achieved in the context of its import/export in conjunction with its biosynthetic pathway is poorly understood.

There is well documented evidence that CT can control its own synthesis by regulating AGAT expression via feedback loop ^11–16^. This mechanism is highly conserved amongst various species, including rats^17–19^, pigs^20^, and chickens^12^.

The conserved CT-mediated feedback loop leads to reduced GAA in healthy adults supplemented with CT^21^. But more importantly, the CT-dependent feedback loop is relevant in pathophysiology and treatment of CT deficiency syndromes (CDS). CDS are inborn errors of CT synthesis and CT transport that are caused by pathogenic variants in *GATM*, *GAMT* and *SLC6A8*^22^. Without detailed knowledge of molecular mechanisms involved, CT-dependent AGAT downregulation is an important part of GAMT deficiency treatment since CT supplementation reduces neurotoxic levels of AGAT-derived GAA in patients with GAMT deficiency^22–24^. That AGAT expression is dynamically regulated by CT levels has more recently been confirmed for CT depletion up-regulating AGAT expression in rodent models of CDS. In response to intracellular CT depletion in CrT deficient mice AGAT protein levels are upregulated in muscle^25^ and kidney^26^, with increased muscle CT synthesis rate^25^, which was, however by far insufficient to compensate the loss of CT transport in the mouse model. Interestingly, a man with CrT deficiency with near absent CT in brain had CT in his muscle that was not significantly different from controls^27^, rising the possibility of compensatory upregulation of AGAT expression sufficient to replenish muscle CT. Regulatory mechanisms involved in the CT-mediated feedback loop, which are successfully utilized for AGAT reduction as important contribution to treatment of GAMT deficiency, may well represent possible therapeutic targets for CrT deficiency^16^, which lacks an efficient treatment so far.

Increased levels of circulating CT results in two-fold or greater reduction in AGAT mRNA, protein, and enzymatic activity^28^. Although the mechanism by which CT regulates AGAT is not fully understood, in vitro translation assays using mRNA isolated from naïve and CT treated mouse kidneys suggest that the repressive effects of CT act on AGAT at a pre-translational level^14^. Cyclocreatine (CycCT) and GAA are both structurally similar to CT and can be transported by the CrT^6,29,30^, but not GAA only CycCT is capable of repressing AGAT expression^31,32^. These observations suggest the existence of an extracellular/intracellular CT/CycCT sensing mechanism that is involved in regulating the expression of AGAT.

Currently, most experiments examining the repressive activity of CT have been performed using animal models^12,18,20^. However, a recent publication by Zhang et al.^32^ showed that CycCT treatment of leukemia cell lines resulted in decreased expression of AGAT, suggesting that cell models could be used to study this regulatory pathway. Thus, we endeavored to establish cell models to elucidate and determine the mechanisms of CT-mediated control of AGAT more easily. Although gene regulation of AGAT, which is physiologically expressed mainly in kidney, pancreas, and some brain cells, must be considered somewhat non-physiological in HAP1 cells, HAP1 cells are among the immortalized human cell lines expressing AGAT (as well as GAMT and CrT) and respond well to CT-mediated downregulation of AGAT expression. HAP1 cells represent a quasi-stable haploid cell line that has been used extensively in CRISPR-Cas9 based genome editing experiments to generate specific gene knockouts, targeting tagging of genes corresponding to the N- and C-termini of the protein coding region^33^, and in chemical and genetic screening campaigns^34^ (and reviewed in^35^). As such, we have used CRISPR homology- mediated repair^36^ to tag AGAT with nanoluc luciferase (NLuc) in HAP1 cells to quantify and monitor the changes in AGAT-NLuc expression with increasing concentrations of extracellular CT. These reporter lines have also been used to examine the role of the CrT in facilitating CT-mediated control of AGAT. By pairing the change in AGAT-NLuc expression with intracellular CT levels, we suggest the existence of an intracellular sensor that may be involved in mediating the CT-dependent feedback loop.

## Results

### Tagging the endogenous *GATM* gene with luciferase to quantitatively monitor the effect of creatine on AGAT expression.

**Figure 1:**
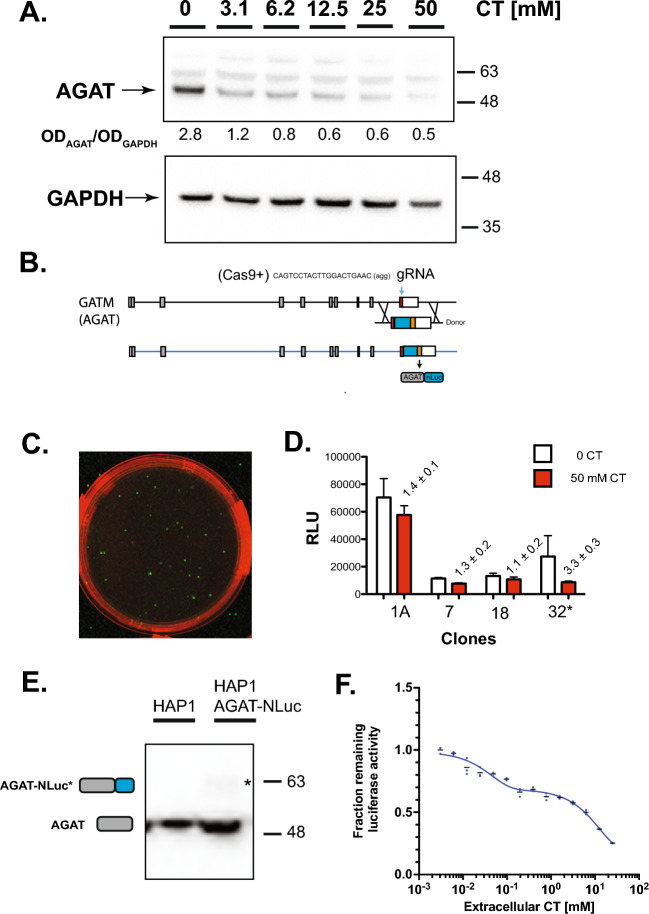
Generation and preliminary characterization of a HAP1 CrT^WT^ AGAT NanoLuc luciferase (NLuc) reporter line. (**A**) Western blot of creatine (CT) treated WT HAP1 cells showing a decrease in AGAT expression levels with increasing extracellular CT (uncropped blots see supplementary Figure S1). (**B**) Schematic of donor plasmid and guide RNA location used to insert a NLuc cDNA after the coding region in exon 9 of AGAT at c-terminus. White squares correspond to 5' and 3’ non-coding regions of exon whereas gray shaded squares represent coding regions. Sequence of gRNA is shown above exon 9 (red box) with approximate position denoted by blue arrow. Schematic of donor plasmid containing NLuc cDNA (blue box) and protein destabilization domain (orange box) are shown below exon 9. Expression from the modified *GATM* gene is expected to result in a larger AGAT-NLuc fusion protein with luciferase activity towards the substrate Coelenterazine H that can be monitored using a luminometer. (**C**) Identification of NLuc expressing (luminescence shown in green) colonies following transfection of HAP1 cells with the *GATM* gRNA and donor plasmid and addition of Coelenterazine H substrate to live cells and imaged under the chemiluminescent channel on a BioRad Chemidoc. (**D**) Identification of NLuc clones showing reduced luminescence (luciferase activity) when grown for 24h in media containing 50 mM CT (red bar). Numbers above red bar refer to the fold-reduction in luciferase activity in the presence of CT (n = 3, mean ± SD). Clone 32 (asterisk) that showed the greatest response to CT was selected for further analysis. (**E**) Characterization of CT responsive AGAT-NLuc reporter clone 32 by western blotting: position of larger AGAT-NLuc fusion protein denoted by arrow adjacent to the corresponding band on the western blot. Bars to the right show position of molecular weight standards in kilodaltons (uncropped blot see supplementary Figure S2). (**F**) Response of AGAT-NLuc reporter to increasing concentration of extracellular CT. Luciferase activity (fraction remaining) at a given CT concentration is normalized to luciferase activity (relative luminescence units [RLU]) in growth media that has not been supplemented with additional CT (n = 3, mean ± SD).

HAP1 cells express readily detectable levels of the three important components of the CT-synthesis/transport pathway, including AGAT, GAMT and CrT^37^. We have demonstrated that AGAT protein levels decrease in a dose dependent manner with increasing concentration of extracellular CT (Fig. 1A). To quantitatively monitor the effect of CT on AGAT expression we added NLuc in-frame to the end of the predicted C-terminus of AGAT with CRISPR-mediated homology directed repair (HDR) using a donor template (Fig. 1B). There are isoforms of AGAT differing at the C-terminus that are derived by alternative splicing at exons 8 and 9. We chose to add NLuc at the end of exon 9 as it is the last exon in the predominant isoform expressed in tissues and cell lines^37,38^. We selected single cell colonies that expressed Nluc (Fig. 1C). From 10 NLuc positive clones, one clone was selected for further study that responded to CT with over three fold reduction in luminescence (Fig. 1D). PCR experiments confirmed that NLuc has been inserted at one of the two copies of the *GATM* gene. Western blotting confirmed the presence of a band corresponding to WT AGAT and a larger 60 kDa band corresponding to the AGAT-NLuc fusion protein (Fig. 1E). Sequencing confirmed that NLuc was in-frame with the C-terminal residues of NLuc and the DNA sequence corresponding to the C-terminus at the other allele was identical to the WT sequence (data not shown). The AGAT-NLuc reporter line responded with suppression by CT in a dose dependent manner (Fig. 1F). The decrease in AGAT-NLuc luciferase activity parallels the decrease in AGAT protein (supplemental Figure S3 A,B,C,D,E). The potential of direct inhibition of NLuc enzymatic activity by CT had been excluded (Supplementary Figure S3F).

### AGAT-NLuc expression responds to extracellular creatine with a double decay curve

**Figure 2:**
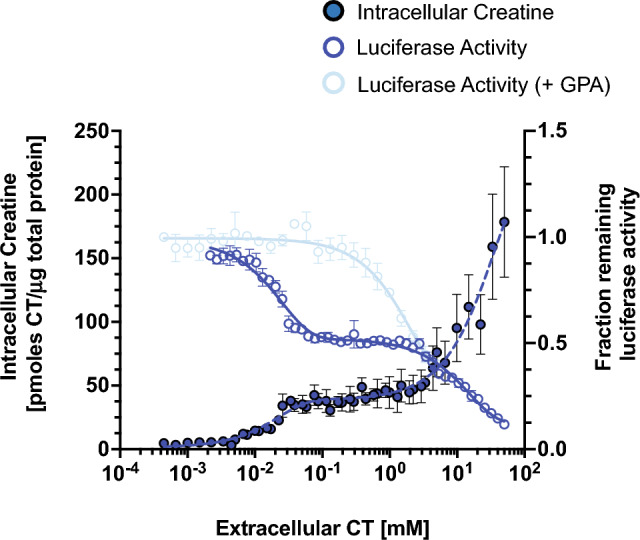
Change in AGAT-NLuc activity and intracellular creatine (CT) with increasing extracellular CT. The best CT responding clone 32, was grown for two days in media containing dialysed media, followed by 24 h in media containing dialysed FBS supplemented with increasing amounts of CT (n = 4, mean ± SD). Fraction remaining luciferase activity shown on the right axis is derived from the Relative Luminescence Units (RLU) at a given extracellular CT concentration normalized to RLU in control media that has not been supplemented with CT. The decrease in AGAT-NLuc activity with increasing extracellular CT displays a two-phase decay curve (solid line, open circle, right axis). Correspondingly, the increase in intracellular CT with increasing extracellular CT displays a two-phase exponential increase (dotted line, filled circle, left axis) (n = 3, mean ± SD). Intracellular CT levels were determined by the ninhydrin-based fluorometric assay. Only a single-phase AGAT-NLuc activity decay curve (light blue solid line, open circles, right axis) was observed following treatment of AGAT-NLuc cells with CT and 5 mM guanidinopropionic acid (GPA), a known CrT inhibitor (n = 3, mean ± SD).

We determined that normal cell growth media containing 10% fetal bovine serum (FBS) had CT at a concentration of approximately 24 μM (determined by LC/MS/MS). To assess the expression of AGAT in the absence of CT, we cultured the AGAT-NLuc reporter cell line in media containing 10% FBS that had been dialyzed (6–8 kDa cut-off, from here on referred to as ‘dialyzed media’). To ensure that the media was CT-free, LC–MS/MS was performed which showed that the concentration of creatine was below the detection limit (< 0.01 μM). In the previous experiment, cells cultured in normal media followed by CT treatment implied a two-phase decay curve (Fig. 1F). Cells cultured in dialyzed media as well as treatment with greater range of CT concentrations confirmed such two-phase decay curve. The first phase appears between 1–100 μM and the second phase is between 1 and 50 mM (Fig. 2). Fitting the dose response data to a two-phase decay curve resulted in two predicted inflection points at 0.02 mM and 2 mM. The first inflection point value is close to the published experimentally derived Km for CrT of 22–52 μM^7,8,39,40^, emphasizing that functional CrT is crucial for the intracellular response to extracellular CT exposure in concentrations less than 100 μM.

**Figure 3:**
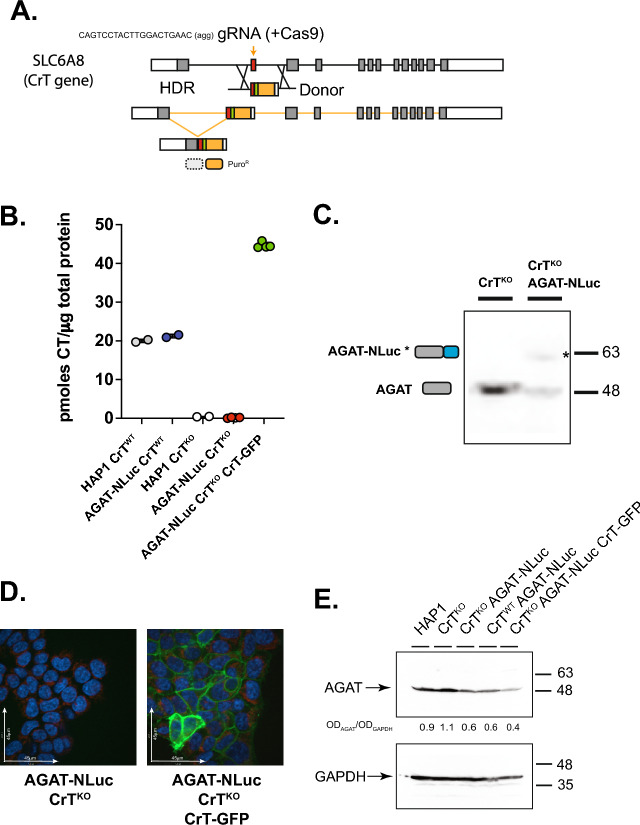
Generation and characterization of creatine transporter (CrT) knockout HAP1 cells and derivatives expressing AGAT-NLuc and a WT CrT-GFP fusion. (**A**) Donor vector used to insert T2A (green bar) puromycin coding sequence (orange bar) into the coding region of exon 2 (red bar) of human *SLC6A8* gene and location of gRNA (orange arrow) used to target exon 2 and primers used to validate puromycin resistant knock-in clones. The cartoons below the donor vector show the result of the integrated donor DNA, spliced product following transcription and expected translation products, respectively. The resulting translation products are predicted to consist of a non-functional prematurely terminated *SLC6A8* N-terminal peptide and puromycin N-acetyl transferase enzyme conferring puromycin resistance. (**B**) LC/MS/MS determined CT levels in the selected puromycin resistant clone bearing an insertion in exon2 of *SLC6A8* are significantly reduced in comparison to parental HAP1 cells (individual replicates are shown). (**C**) The genome editing procedure outlined in Fig. 1A was used to tag the c-terminus of AGAT with NLuc in the HAP1 CrT^KO^ line. Western blot of lysate from AGAT-NLuc CrT^KO^ clone probed with Rabbit antibody against AGAT. Arrow denotes the position of the band corresponding to the larger AGAT-NLuc fusion. AGAT-NLuc CrT^KO^ clone is heterozygous with one allele bearing unmodified *GATM* gene and the other allele containing a NLuc insertion. Bars to the right show position of molecular weight standards in kilodaltons (uncropped blot see supplementary Figure S2). (**D**) Wildtype *SLC6A8* GFP fusion was stably expressed in AGAT-NLuc CrT^KO^ clones following transfection with a plasmid bearing a WT human CrT-GFP fusion cDNA under the control of a CMV promoter. Spinning disk confocal image of AGAT-NLuc CrT^KO^ cells stably expressing a CrT-GFP (right panel) compared to the parental line (left panel); GFP fluorescence in green and nuclei stained with Hoescht in blue. (**E**) Western blot of lysate from HAP1, CrT^KO^, CrT^KO^ AGAT-NLuc, CrT^WT^ AGAT-NLuc, and CrT^KO^ AGAT-NLuc CrT-GFP cells probed with either AGAT Ab or GAPDH Ab. The OD_AGAT_/OD_GAPDH_ values between the two panels represent intensity of AGAT band normalized to the intensity of the GAPDH band. Position of molecular weight markers are shown to the right of the panels (uncropped blots see supplementary Figure S3).

To determine the role of high-affinity CT uptake mediated by CrT on cellular response to extracellular CT concentration less than 100 μM, we generated an AGAT-NLuc reporter line, HAP1 CrT^KO^ AGAT-NLuc, in which both copies of *SLC6A8* were mutated via insertion of a puromycin coding cassette inserted in frame in exon 2 by Cas9-mediated HDR using a gRNA targeting exon and the donor template as shown in Fig. 3A. We used PCR to confirm that the mutants were homozygous. Most importantly, the homozygous mutants had significantly reduced intracellular CT levels as determined by LC/MS/MS (Fig. 3B). We also inserted an NLuc reporter in one allele in frame at the C-terminus of AGAT in exon 9 using the same approach as described above and in Fig. 1. Western blotting confirmed the presence of a band corresponding to WT AGAT in the HAP1 CrT^KO^ line and a larger 60 kDa band corresponding to the AGAT-NLuc fusion protein in the HAP1 CrT^KO^ AGAT-NLuc line (Fig. 3C).

To restore CT uptake in the HAP1 CrT^KO^ AGAT-NLuc line we transfected the cell line with a CrT-GFP fusion plasmid under the control of a CMV promoter and selected clones stably expressing the CrT-GFP fusion transgene. Confocal imaging showed the presence GFP positive cells with green fluorescence limited to the cellular periphery (Fig. 3D), and LC/MS/MS confirmed that CT uptake had been restored in HAP1 CrT^KO^ AGAT-NLuc CrT-GFP cells (Fig. 3B).

In Fig. 3E, the expression of AGAT is shown in all 5 cell lines used for this work. We noted the AGAT abundance was ca. 50% in all 3 NLuc reporter cell lines compared to the AGAT protein in HAP1 WT and HAP1 CrT^KO^ what could be explained by the presence of a protein destabilization sequence in the Nanoluc luciferase reporter plasmid.

### High-affinity creatine uptake enables intracellular response, a.k.a. AGAT-NLuc repression, at low/physiological extracellular creatine concentration. AGAT-NLuc expression inversely correlates with intracellular creatine concentration

**Figure 4:**
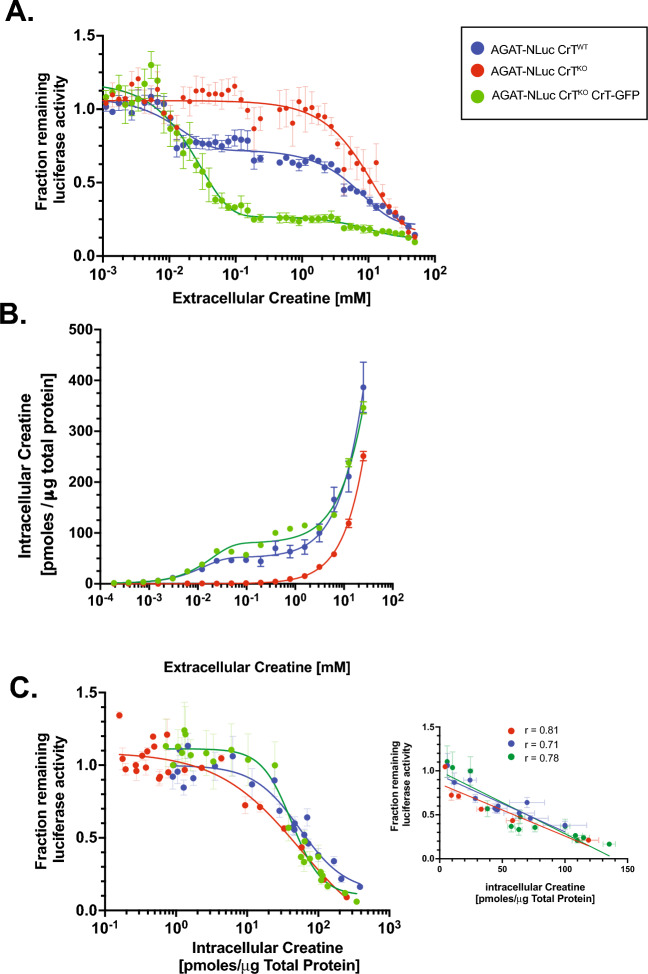
Comparison of AGAT-NLuc response and intracellular creatine (CT) levels with increasing extracellular CT in the presence and absence of the CT transporter (CrT). All cells were grown in media containing dialysed FBS two days prior to 24 h treatment with media containing dialysed FBS supplemented with increasing dose of CT. (**A**) Response of AGAT-NLuc activity to increasing concentrations of extracellular CT in CrT^WT^, CrT^KO^ and CrT^KO^ CrT-GFP lines (n = 3, mean ± SD). (**B**) Change in intracellular CT with increasing concentrations of extracellular CT in CrT^WT^, CrT^KO^ and CrT^KO^ CrT-GFP lines (n = 3, mean ± SD). 100,000 cells were treated in triplicate at each dose of CT. Approximately 25% of the cells were used to determine CT levels using the ninhydrin fluorescence assay. (**C**) Quantifying change in intracellular CT versus AGAT-NLuc activity in CrT^WT^, CrT^KO^ and CrT^KO^ CrT-GFP lines derived from data presented in Figs. **A** and **B**. The small graph in the inset shows the relationship between luciferase activity versus intracellular CT plotted on a linear scale. Correlation coefficients for each of the curves are shown above.

Treating cells with increasing concentrations of extracellular creatine, we observed AGAT-NLuc repression in HAP1 CrT^KO^ AGAT-NLuc cells beginning at approximately 1–2 mM CT, while wild type cells and cells overexpressing CrT-GFP responded to extracellular CT already at 0.01 mM CT (Fig. 4A). Compared to HAP1 CrT^WT^ AGAT-NLuc cells where a two-phase decay in relative luciferase activity was seen with a plateau between 0.05 and 2 mM, only a single-phase decay starting at 2 mM was observed in the HAP1 CrT^KO^ AGAT-NLuc cells. Furthermore, the AGAT-NLuc response to CT at low extracellular concentrations could be restored upon overexpression of a CrT-GFP fusion protein (Fig. 4A). The combined results for the CrT^KO^ reporter lines with and without CrT overexpression strongly suggest that CrT is crucial in the response to low or physiological concentrations of extracellular CT, also confirming our findings mentioned above (Fig. 2).

Next, we set out to investigate the relationship between intracellular and extracellular CT. We used a 96-well adapted CT fluorescence assay for intracellular CT quantification^41^. In the HAP1 CrT^WT^ AGAT-NLuc cells, there was a linear increase in intracellular CT from 0.001 to 0.1 mM extracellular CT followed by a plateau and ending with another linear increase from 1–50 mM extracellular CT (Fig. 4B). In contrast, in the HAP1 CrT^KO^ AGAT-NLuc line, an increase in intracellular CT was not observed in the micromolar range of extracellular CT and could only be detected from 1 mM upwards (Fig. 4B). The increase of intracellular CT at lower concentrations of extracellular CT was restored in the CrT^KO^ AGAT-NLuc CrT-GFP overexpressing cells (Fig. 4B), allowing the conclusion that the CT uptake at lower extracellular CT concentrations is dependent on the expression of CrT and is negligible without this high-affinity transporter.

Plotting the change in relative AGAT-NLuc expression versus intracellular CT concentration for all the examined cell lines – CrT^WT^ AGAT-NLuc, CrT^KO^ AGAT-NLuc, and CrT^KO^ AGAT-NLuc CrT-GFP – revealed a similar clear dose dependent and saturable decrease in AGAT-NLuc expression with increasing intracellular CT concentration in all cell lines (Fig. 4C). In fact, the curves were reminiscent of sigmoidal inhibitory curves with an approximate IC50 of 52 pmol CT/μg total protein. Furthermore, in the range between 5 and 100 pmol CT/μg total protein, there was an inverse linear relationship of intracellular CT with NLuc activity that was unchanged whether CrT activity was present, knocked out or overexpressed. Irrespective of the mechanism by which CT entered the cells, increasing intracellular concentrations of CT determined the degree to which AGAT-NLuc expression was repressed.

### Application of the AGAT-NLuc reporter cell lines for assessing CrT kinetics

**Figure 5:**
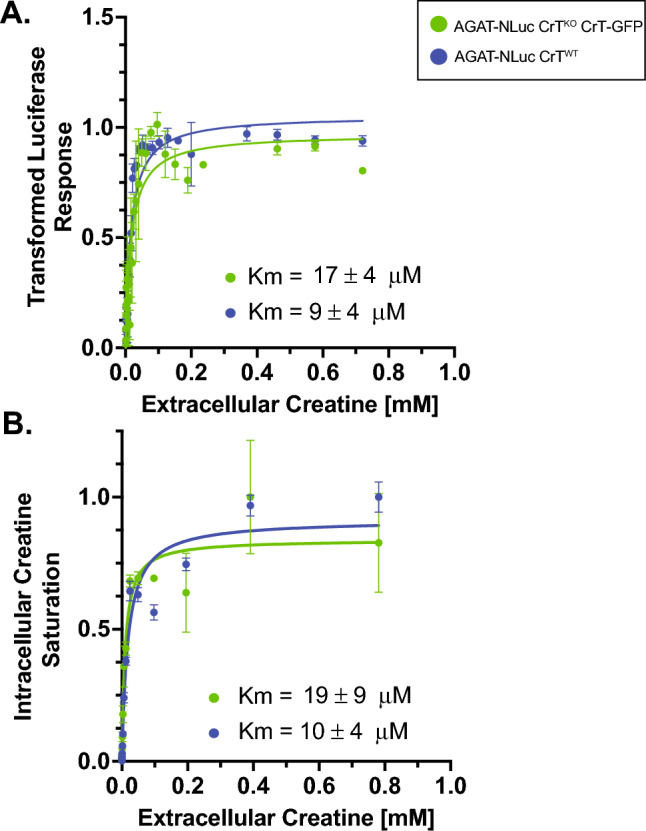
Comparison of creatine (CT) uptake kinetics attributed to CT transporter (CrT) derived from intracellular CT levels and AGAT-NLuc activity in response to extracellular CT levels (1–1,000 μM range). (**A**) Decay curves of AGAT-NLuc activity at less than 1 mM in CrT^WT^ and CrT^KO^ CrT-GFP lines were transformed to using the Eq. 1-(Fi-min(F))/(max(F)-min(F)), where F represents the fraction remaining AGAT-NLuc activity to generate a Michaelis–Menten curve. AGAT Nluc activity values are corrected for CT uptake by the CrT independent CT uptake using the data from AGAT-NLuc activity values in the CrT^KO^ line. Extracted Km values for both cell lines are shown under the curve. (**B**) Michaelis–Menten curves derived from intracellular CT levels in CrT^WT^ and CrT^KO^ CRT-GFP lines at extracellular CT levels less than 1mM. Values are corrected for CT uptake by the CrT independent CT uptake using the data from intracellular CT levels in the CrT^KO^ line. Extracted Km values for both cell lines are shown under the curve (n = 3, mean ± SD).

Given the direct response of the AGAT-NLuc signal to changing intracellular CT concentrations and its readability, we wondered whether one could apply this model as a biosensor to determine CT uptake kinetics. Using the readouts of either the remaining NLuc luminescence or the intracellular CT concentration determined with the fluorometric assay, we established Km values for high-affinity CT uptake at 9 ± 4 μM (AGAT-NLuc signal, Fig. 5A) and 10 ± 4 μM (CT signal, Fig. 5B) after correction for the linear and unsaturable uptake process. Our Km values obtained using either NLuc luminescence or intracellular CT as readouts are lower but in close agreement to the range of published values (22–52 μM)^7,8,39,40^.

The magnitude of interference of the CrT independent CT uptake on CrT kinetics was deemed negligible considering the strong separation by orders of magnitudes of CT concentrations involved in each of the uptake processes. Despite that, we corrected for the interfering CrT independent, unsaturable process by subtracting the AGAT-NLuc activity and intracellular CT values in the CrT^KO^ AGAT-NLuc line from those in the CrT^WT^ and CrT^KO^ AGAT-NLuc CrT-GFP lines. As expected, the contribution of this CrT independent process increased the apparent KM by only an average of 10% .

### Application of the AGAT-NLuc reporter cell lines for assessing activity of the CT-mimetic cyclocreatine on CrT-mediated uptake and AGAT-NLuc repression

**Figure 6:**
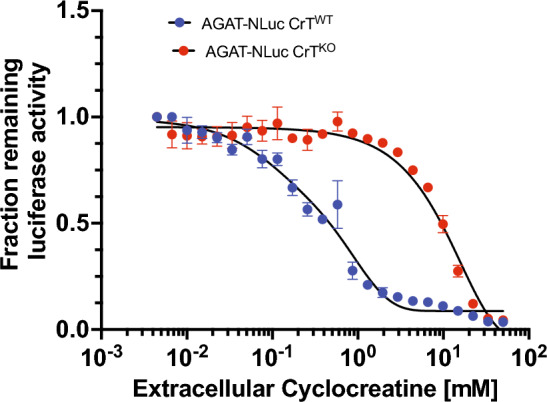
Cyclocreatine (CycCT) decreases AGAT-NLuc expression in a creatine transporter (CrT) dependent and CrT independent manner. AGAT-NLuc in CrT^WT^ or CrT^KO^ cells grown in media containing dialysed FBS were subsequently treated (for 24 h) with media containing dialysed FBS supplemented with an escalating dose of CycCT. Change in luminescence with CycCT was normalized to luciferase activity in cells grown in media lacking CycCT (n = 4, mean ± SD).

We have evaluated the ability of cyclocreatine (CycCT), a ringed-mimetic of CT to serve as a CrT substrate using the CrT^WT^ AGAT-NLuc and CrT^KO^ AGAT-NLuc lines. Similar to the response to CT, we observed a two-phase activity decay curve after exposure to CycCT that was present in the CrT^WT^ AGAT-NLuc line but not in the CrT^KO^ AGAT-NLuc line (Fig. 6). The apparent IC50 of extracellular CycCT, 180 μM, extracted from the first phase of the decay curve, is a magnitude greater than the IC50 for extracellular CT (17–24 μM). Comparison of the effects of escalating dose of extracellular CycCT on AGAT-NLuc activity in CrT^WT^ versus CrT^KO^ in Fig. 6 demonstrated that the effect of CycCT at extracellular concentrations less than 0.8–1mM is dependent on CrT function.

**Figure 7:**
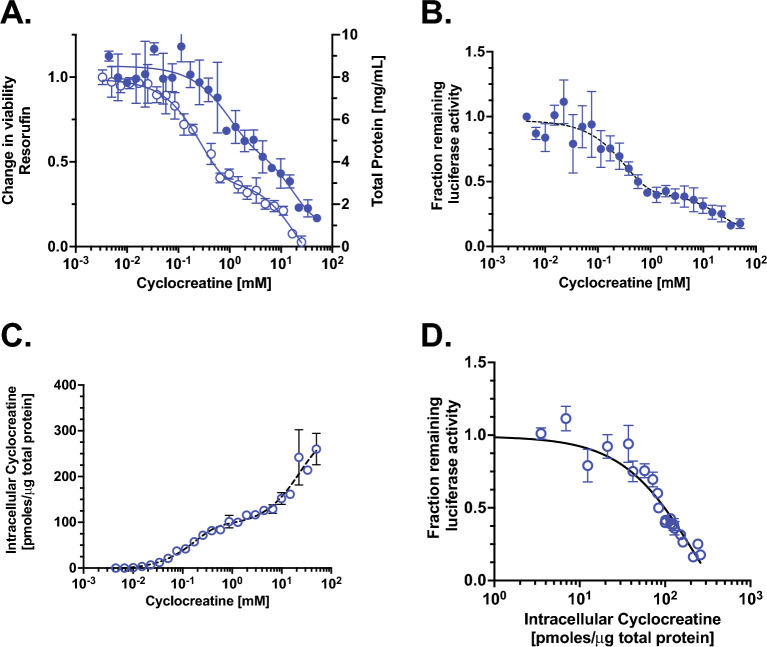
Decrease in AGAT-NLuc expression shows an inverse linear dependence on intracellular Cyclocreatine (CycCT) levels. (**A**) Total protein concentration [mg/mL] (right y-axis) in lysate derived from AGAT-NLuc CrT^WT^ treated with an escalating dose of CycCT (n = 2, mean ± SD). Viability was determined using resorufin fluorescence (ex. 570 nm, em. 590 nm) and expressed as a change in fluorescence (left y-axis) normalized to the fluorescence of cells grown in unsupplemented normal media (n = 3, mean ± SD). Viability of treated cells decreases below -3 SD at concentrations greater than 100 μM. (**B**) The decrease in AGAT-NLuc expression (normalized to total protein in lysate) shows a two-phase decay with increasing extracellular CycCT (n = 4, mean ± SD). (**C**) Intracellular CycCT determined by LC/MS/MS and normalized to total protein increases in two phases with increasing dose of extracellular CycCT (n = 2, mean ± SD). (**D**) Change in luciferase activity (from panel B) plotted versus change in intracellular CycCT (from panel C). The change in AGAT-NLuc activity varies in an inverse linear manner with increasing intracellular CycCT.

Next, we investigated the CycCT uptake kinetics. The intracellular CycCT concentration (determined by LC/MS/MS) responded to increasing concentrations of extracellular CycCT in a bi-phasic curve with a plateau at approximately 1 mM extracellular CycCT, followed by further increase that was not saturable (Fig. 7C). The apparent Km for the first, and saturable phase of CycCT uptake was ca. 200 μM (compared to 9–10 μM for CT uptake).

To assess the effect of intracellular CycCT on AGAT-NLuc repression, cytotoxic effects of CycCT must be considered. We found reduced viability of cells based on resorufin fluorescence and decrease of total protein in cell lysates treated with extracellular CycCT above ca. 100 μM and 800 μM, respectively (Fig. 7A). CycCT effects on AGAT-NLuc repression we therefore normalized total protein.

By correlating the extracellular CycCT concentration with the relative AGAT-NLuc luciferase activity, we obtained a decay curve with an inflection point at 800 μM extracellular CycCT (Fig. 7B). Correlating the AGAT-NLuc response to intracellular CycCT concentration revealed an inverse linear relation (Fig. 7D) similar to that of CT (Fig. 4C). However, the apparent IC50 of 210 pmol CycCT/μg total protein is 4 times greater than that for CT (52 pmol CT/μg total protein).

## Discussion

For the first time, we have demonstrated CT- mediated downregulation of AGAT in a cell model system. Our system can be used to monitor the degree of AGAT expression in response to changes in extra- and intracellular CT easily and quantitatively.

Secondly, we have demonstrated that CT-mediated downregulation of AGAT is responding to the operation of both an CrT dependent and an CrT independent mechanism to uptake extracellular CT into the cell, and therefore is independent of CT entry route.

Lastly, although the AGAT expression responds to extracellular CT, the decrease in expression correlates directly and in proportion with the intracellular CT concentration, which implies the existence of an intracellular CT sensor that controls expression of AGAT. Although we don’t want to speculate about the potential mechanism responsible for the CT-mediated AGAT downregulation, we can imagine the existence of a post-transcriptional control via ribosomal functioning. Such mechanism were ribosomes act as L-tryptophan sensors has been observed in the negative feedback loop of L-tryptophan synthesis in *E. coli*^42^.

### A convenient cell model to study CT-mediated downregulation of AGAT expression

We have used CRISPR generated NLuc reporters tagged at the C-terminus of AGAT to monitor the effect of extracellular CT on expression from the *GATM* locus in the context of WT or null mutant CRT in HAP1 cells. Considering isoforms of AGAT^43^, specifically the ones that differ at the C-terminus derived by alternative splicing at exons 8 and 9, we chose to add NLuc at the end of exon 9 as it is the last exon in the predominant isoform expressed in tissues and cell lines^37,38^. The CT-mediated changes in AGAT expression determined by western blotting parallel the changes in AGAT-NLuc expression using luciferase activity, suggesting NLuc activity of the fraction of NLuc-tagged AGAT protein nicely reflects the overall AGAT protein content and can be used as valid readout for regulation of AGAT expression. Furthermore, the use of the NLuc reporter is advantageous since changes in AGAT-NLuc luciferase activity can be more simply and easily quantitatively monitored in a 96-well format in comparison to western blotting, making it a valid tool for high-throughput screening for AGAT regulators.

One potential drawback of our approach is of course the non-physiological myeloid cancer background of HAP1 cells. The technical advantage of this line’s near haploid karyotype, simplifying genetic manipulations (e.g. CRISPR based gene-editing) does not effect the two genes manipulated in this study: The first one, AGAT, is among the few diploid chromatin regions in this cell-type and the second one, CrT, is an X-chromosomal gene that is haploid (or more precisely hemizygous) in any male derived cell line (like HAP1) anyway. So, gene regulation of AGAT, which is physiologically expressed mainly in kidney, pancreas, and some brain cells, must be considered somewhat non-physiological in HAP1 cells. Nevertheless, HAP1 cells are among the immortalized human cell lines expressing AGAT (as well as GAMT and CrT) so regulatory mechanisms may be identified here. However, in the long run this needs to be somehow linked to patho-physiologically relevant (brain) cells (like neurons, oligodendrocytes, and glia cells).

### Involvement of CrT dependent and CrT independent pathways in CT-mediated downregulation of AGAT expression

The AGAT-NLuc reporter cell line in combination with CRISPR-mediated mutagenesis has enabled us to study molecular aspects of the CT feedback loop, here specifically the role of CT uptake and location of CT sensing. The biphasic response in decrease of AGAT-NLuc expression in response to increasing extracellular CT confirmed previous findings of the existence of two separate CT uptake mechanisms: a high affinity, saturable CrT dependent uptake, and a low affinity, unsaturable CrT independent uptake. The latter likely being irrelevant under physiological conditions. We used CRISPR to directly demonstrate the involvement of CrT in CT-mediated downregulation at lower, a.k.a. physiological concentrations of CT. Knocking out the CrT, the high affinity uptake could be restored by re-introducing a cDNA coding for a WT-CrT-GFP fusion.

It was possible to deduce the Km values directly by measuring intracellular CT concentration or indirectly (and more easily) by recording the AGAT-NLuc response to extracellular CT. The obtained Km values for CT uptake were similar for both the endogenous CrT in the WT AGAT-NLuc cell line and the exogenous CrT-GFP expressed in the CrT Knockout AGAT-NLuc cell line, though the apparent Km seems to be twice as high in the CrT-GFP cells, potentially caused by GFP slightly interfering with the CrT affinity for CT. The Km of the endogenous HAP1 CrT (9–10 μM) was lower, but within the range of Km values obtained for the rat and human CrT, 22–39 μM ^7^, 30 μM^8^, while others observed slightly higher Km values, 48 μM^39^ and 52 μM^40^. With the CrT knockout, we were able to exclude the CrT independent CT uptake contribution, that may explain why we observed higher affinity results than others. When including both the high- and the low affinity mechanism our calculations would result in an apparent Km of 17–24 μM that is much closer to the reports in the literature.

We were also able to determine the Km values for CycCT uptake by the CrT, 121 and 229 μM, using AGAT-NLuc activity and intracellular CycCT concentration, respectively. These values are in line with the Km value of 188 μM determined using uptake of^14^C labelled CycCT in Hek293 cells overexpressing CrT^29^. In the future, the AGAT-NLuc CrT knockout reporter cell line could be used to easily measure transport activity of other CT mimics as well as to assess the severity of CrT deficient patient derived mutations by analysing the CT transport activity of overexpressed CrT mutants.

Although the CrT independent pathway for CT uptake has been previously noted^44^, the components of this pathway have yet to be identified. Previous observations suggested that the CrT independent pathway involves passive/facilitated diffusion mechanism. The linearity of decrease in AGAT-NLuc response and the increase in intracellular CT with increasing extracellular CT concentrations is consistent with a passive/facilitated diffusion mechanism. As an example of facilitated diffusion the GLUT4 transporter enables glucose uptake provided there is an inward diffusion gradient^45^. In the future, our reporter cell-line together with targeted/untargeted CRISPR gRNA screening could be used to study molecular mechanisms, like CT sensing, CT uptake and -export, transcriptional- and posttranscriptional regulation further dissecting molecular details of the CT feedback loop, but also to investigate CT homeostasis at large.

### CT-mediated response correlates directly to intracellular CT levels

Whereas the AGAT-NLuc response to extracellular CT shows a biphasic decay, the response to intracellular CT displays a single-phase decay curve similar to a sigmoidal inhibitory dose response curve. It is possible to extract an IC50 value for the inhibitory effect of intracellular CT on AGAT-NLuc expression. The IC50 of 51 pmol of CT/μg of total protein corresponds to a concentration of 1–2 mM CT using a conversion factor based on the cell volume of HAP1 cells (5.2e^−10^ mL) assuming a sphere with diameter 5 microns and average total protein content per HAP1 cell of 250 pg. Interestingly, this IC50 value overlaps with the intracellular CT concentration of 1.5 mM calculated for half-maximal repression of AGAT activity in chicken liver^46^. The implications of these results are several-fold. First, the direct one-to-one relationship between intracellular CT concentration and AGAT-NLuc response suggest the effect is directly mediated by intracellular CT and implies that there is an intracellular factor (i.e., sensor) that interacts with CT. Secondly, the affinity of CT for the ‘sensor’ is low, given the millimolar concentration of the IC50 value. Lastly, based on these results, under physiological conditions AGAT expression is predicted to be suppressed to at least 50% in cells with functional CrT and intracellular CT concentration of 1–2 mM.

We and others have shown that CycCT can also repress AGAT expression^46,47^. Two other structurally related compounds N-acetimidoylsarcosine and N-ethylguanidinoacetate have been previously shown to act as weak CT-mimetics in terms of their ability to reduce AGAT expression^48^. Compounds such as GAA, guanidinopropionic acid or creatinine which either lack the imino moiety on the amidino group, or the methyl group on the guanidino group, or contain alkyl chain greater than two carbons attached to the carboxylate group are unable to decrease AGAT expression significantly. Although CycCT maybe more membrane-permeable in comparison to CT in terms of its LogP values, its import is still CrT dependent as shown here (Figs. 6 and 7) and by others. Although CycCT can be phosphorylated, phosphocyclocreatine is poorly hydrolyzed and accumulates intracellularly leading to toxicity. Even considering the toxicity of CycCT (Fig. 7) it nonetheless also downregulates AGAT-NLuc expression. We obtained an IC50 value of 150–180 pmol of CycCT/μg total protein, which corresponds to an IC50 value of 4–8 mM for CycCT based on calculated cell volume and intracellular protein concentrations of HAP1 cells. This value is more than three-fold higher than the value obtained for CT. The differing IC50 values for CT and CycCT lend further support for the existence of an intracellular sensing system that interacts with various inducers (CT and CycCT) at different affinities.

Our studies suggest that under physiological conditions in cells and tissues with fully functional CT uptake and high concentration of CT the expression of AGAT must be partially or largely repressed. The variation in intracellular CT levels between different cell types and tissues is large, ranging from 1–3 mM in HeLa, Hek293, HAP1, kidney and liver, 5–20 mM in pancreas and brain to 20–50 mM in selected immune cells and skeletal muscle^3–6^. If there is a similar IC50 value of CT for AGAT repression in these cells and tissues as the one we have established in HAP1 cells it would suggest a supressed CT synthesis in tissues with high CT abundance. In contrast, tissues with low CT, like the kidneys and liver, may respond to the CT feedback loop to much lesser extend since the AGAT expression is not or only slightly repressed by CT under physiological conditions. Therefore, the CT feedback loop dynamically responding to fluctuating CT abundance may be of importance for immune cells and tissues like the muscle that have physiologically high CT contend. The depletion of CT in these cells and tissues, be it through impaired uptake or decreased supply, could trigger lesser AGAT repression and in turn upregulate the CT synthetic rate, of course provided a non rate-limiting GAMT activity.

## Summary

We have developed a cell model that enables the quantitative monitoring of changes in AGAT expression in response to extracellular CT. Downregulation of AGAT expression is directly dependent on intracellular CT. The CT uptake process consists of a CrT dependent, high-affinity, saturable and a CrT independent, unsaturable mechanism. The response dependence on intracellular CT implies the existence of an intracellular low-affinity (millimolar) CT sensing mechanism that is important to maintain CT homeostasis.

## Materials and methods

### Cell lines and reagents

HAP1 cells used to generate the reporter cells were grown from a frozen stock of HAP1 (C859) passage 6 purchased from Horizon Discovery LTD (Cambridge, UK). Iscove’s media (Ref: 098–150, Wisent) supplemented with 10% Fetal Bovine serum (Multicell) was used to grow HAP1 cells. All cells were grown at 37 °C in a 5% CO_2_ humidified incubator. Dialysed FBS was prepared by dialysing 50 mL of FBS against three changes every 2-4 h with 4L of 0.9% NaCL using a 6-8 kDa cut-off membrane (Spectrapor).

### Generation of reporter cell lines

#### NLuc reporter cell lines

A cDNA encoding NLuc (minus the codon starting ATG) was fused in-frame to the last codon of the endogenous AGAT gene using a donor template via CRISPR-mediated homology directed repair in HAP1 cells. The NLuc cDNA also contained a protein destabilization sequence which is found in the Nanoluc luciferase reporter plasmid (pNL1.2 NLUC, Promega Biotech, USA). We included this sequence to ensure that it would be possible to readily see any transcriptional changes especially if AGAT had a very long- (> 24h) half-life. The donor template was assembled by fusing three DNA fragments consisting of 1), intron 8 and exon 9 (left arm-900bp), 2), the coding sequence of NLuc (700bp derived from pnL1.2 (Promega)), and 3), a 500 bp fragment corresponding the 3’ untranslated region of AGAT in exon 9 (right arm). The three fragments were fused together in two steps, by first fusing the left fragment with NLuc (left-NLuc), then gel purification, and finally left-NLuc was fused with right by overlap PCR using KOD polymerase (Millipore). Fragments were gel purified and digested with AscI PacI and cloned into similarly digested pMX vector (Invitrogen). Donor clone pMXex15NLuc was verified by DNA sequencing (GenBank OR492634). Primers used for overlap PCR are listed in Table 1S: Primers (Supplemental Information). Complementary primers (minus PAM underlined) to the guideRNA targeting the sequence CAGTCCTACTTGGAC**TGA**ACAGG and overlapping with stop codon (bold), were phosphorylated, annealed, digested with Bbs I and ligated with PX458 vector digested with Bbs I. HAP1 cells were transfected overnight with equals amounts (250 ng) of gRNAEx15, AGAT donor pMex15NLuc. Next day, transfection media was replaced with fresh media and following two days growth cells were trypsinized, split into two 10 cm plates allowed to adhere overnight. Cells were grown for two weeks to allow for the appearance of single cell colonies. Luminescent colonies (expressing AGAT-NLuc fusion) were identified by first incubating the cells in media containing Coelenterazine H (Regis Technologies Inc. (IL, USA)) at 5 µg/mL followed by visualization of luminescent colonies using a Biorad Chemidoc XRS imager. Media was replaced with 5 mL trypsin and individual luminescent clones (previously located using a marked fluorescent grid on the back of the tissue culture plate) were picked under an inverted microscope using p200 pipette man. Clones were expanded and tested for response to CT. Those clones that showed decreased luminescence in the presence of 50 mM CT, were further expanded for confirmation of targeting by PCR and sequencing of the products. Two clones were identified and clone ‘C8 32.4’ was used for subsequent experiments since its sequence (GenBank OR492632) matched exactly the sequence of the desired targeted AGAT-NLuc fusion sequence.

#### CrT^KO^ AGAT-NLuc reporter line

CrT^KO^ HAP1 cells were generated by insertion of a puromycin cassette into exon2 of the human CrT gene (*SLC6A8*) by CRISPR-mediated homology directed repair using a donor plasmid consisting of a cDNA coding for puromycin and a polyA addition signal flanked by 1000bp 5’ upstream of exon 2 and 800 bp downstream of exon2 centred on the exon2 gRNA. The start codon of the puromycin was place in frame with a T2A translation re-initiation signal that was also in frame with the CrT codon in exon 2. The right homology arm, puromycin cassette and left homology arms were fused together via golden gateway cloning using BsPI (NEB) flanking adapters and cloned into pMX using AscI and PacI sites to generate the donor plasmid pMXCrTDonoreEx2PuroPA. HAP1 cells were co-transfected with the gRNA (px458CrTEx2) and the donor plasmid (pMXCrTDonoreEx2PuroPA) at a 1:1 ratio (0.5ug:0.5ug) using GeneJet transfection reagent. Following three days culture, puromycin was added to 2ug/mL and puromycin resistant colonies were selected for over a 14-day period. Colonies were picked, expanded and genomic DNA prepared using BioBasic Genomic DNA extraction kit. Primers to either side of the insertion as well as outside the margins of the right and left homology arms were carefully designed to be specific for the functional CrT gene (*SLC6A8*) on the X-chromosome but not the two copies of the CrT pseudogene *SLC6A10P* of chromosome 16. Insertions of the puromycin gene in exon2 of *SLC6A8* were verified by sequencing of the amplified products (data not shown). Two clones (S5 and S2) homozygous for the insertions were selected for tagging of the AGAT gene with NLuc using the protocol described above for generating the CrT^WT^ AGAT-NLuc reporter clones. Ultimately two clones from each of the parental CrT^KO^ lines (S2 and S5) were selected for analysis. Clone S5 (CrT^KO^ AGAT-NLuc, GenBank OR492633) which is also heterozygous for an in-frame insertion of NLuc at the C-terminus of AGAT was used in all experiments described here-in.

#### Generation and characterization of CrT^KO^ AGAT-NLuc CrT-GFP reporter line

To generate a CrT^KO^ AGAT-NLuc clone stably expressing the WT CrT-GFP fusion, the CrT^KO^ AGAT-NLuc cells were transfected (GenJeT) with a plasmid bearing the CrT cDNA (BC012355 – from CCSB Human ORFEOME collect hORFeome V5.1) corresponding to the full length *SLC6A8* open reading frame (ORF) fused in-frame to GFP via gateway cloning into the pcDNADEST47 C-terminal Cycle 3 GFP vector (SPARC cDNA cloning facility https://lab.research.sickkids.ca/sparc-drug-discovery/services/molecular-archives/sparc-vector-information/). Single cell colonies expressing GFP were isolated and expanded, herein referred to as AGAT-NLuc CrT^KO^ CrT-GFP. To image clones, cells were plated onto 96-well plates, treated with Hoescht 3773 for 1h at 37 °C to stain nuclei. Cells were imaged on a Quorum Spinning Disk Confocal microscope (Olympus IX81) under a 60x/1.27NA water objective equipped with Hamamatsu C9100-13 EM-CCD camera and Spectral Borealis lasers at 405 nm (50 mW) and 491 (50 mW) with Emission Filter Wheels: 447/40 and 515/40 for Hoescht and GFP, respectively.

### Determination intracellular CT levels—Ninhydrin-based fluorescence assay

The intracellular CT fluorometric assay is a modification of the assay and protocol described by Conn^41^. We established this method to readily determine intracellular CT concentrations in large number cell experiments. HAP1 cells expressing AGAT tagged at the C-terminus with NLuc were grown for two days in Iscove’s media supplemented with 10% dialysed FBS. Subsequently 100,000 cells were seeded into 12-well plates. The following day, media was removed and replaced with media containing different concentrations of CT. Growth media used to prepare different dilutions of CT contained 10% dialysed FBS. CT levels in the based media were below the limits of detection by LC-MSMS (< 0.1 µM). Two dilution series were prepared one set involving a 24-step serial 1.25 dilution starting at 100 mM and another one starting at 10 mM. Cells were treated with an equal volume of CT dilution series in triplicate overnight (24h). The next day, media was removed, and cells were washed with 2mls PBS three times followed by scraping of the cells into 1mL PBS. Cells were pelleted by centrifugation in a microfuge for 1 min at 15,000 rpm at room temperature. The pellet was frozen at -80°C. Frozen pellets were first thawed and then resuspended in 200 µL distilled water followed by sonication for 10s. The lysate pelleted for by centrifugation for 15 min at 4000 rpm at room temperature. The supernatant (40 µL) was removed and aliquoted into 8-well PCR tubes. To each tube was added 160 µl ethanol, followed by incubation for 15 min at − 20 °C and pelleting of protein by centrifugation in 4500 rpm for 15 min at 4 °C. The supernatant (80µl) was aliquoted into a 96-well polystyrene plate. Each plate contained a CT standard dilution series (80 µL starting at 500 µM, 7 step 2 × dilution and water blank). To each sample was added 40 µL of 1% w/v Ninhydrin (in 80% Ethanol) followed by 40 µL of 10% w/v KOH (in 80% Ethanol). Two fluorometric readings were acquired one immediately after addition of the reagents and the second one after a 60 min incubation at room temperature (plates were covered with a plastic film to prevent evaporation). Readings were performed on a Molecular Devices M2 spectrofluorometer with excitation and emission settings at 310 nm and 510 nm (cut-off at 495 nm), respectively. A CT standard curve was included with each plate.

Sample standard curves for CT, GAA and Arg are highlighted in supplemental Figure S4A,B. CT levels using this assay are linear from 10 to 1,000 μM. Although the ninhydrin-based fluorescence assay been previously used to quantify intracellular CT, ninhydrin also reacts with GAA and Arg to produce a fluorescent product. However, as determined by LC/MS/MS, intracellular levels of Arg (1.4 pmol/μg total protein) and GAA (0.006 pmol/μg total protein) are approximately 10 and 2000-fold lower than CT levels (13.6 pmol/μg total protein) in HAP1 cells grown under normal conditions (Iscoves media 10% FBS, 37°C, 5% CO_2_). Comparing the fluorescence intensity of the fluorescent products, when ninhydrin is reacted with CT, Arg or GAA, the latter two compounds yield fluorescent products that have a fluorescence intensity at least tenfold lower compared to CT at the same concentration (supplemental Figure S4A,B). The significantly lower basal intracellular concentrations of Arg and GAA compared to CT in addition to the fact that the fluorescent products derived from Arg and GAA are significantly less intense compared to that produced by CT strongly suggest that intracellular Arg and in particular GAA do not significantly contribute to the total fluorescence produced by ninhydrin. The majority of changes in fluorescence in this assay can be ascribed to changes in intracellular abundance of CT. We have compared intracellular values for CT determined using the ninhydrin fluorescence versus those determined by LC/MS/MS and both values show a high degree of correlation (supplemental Figure S4C). Intracellular CT levels determined by the ninhydrin assay and LC/MS/MS are linear from 0.1 pmol CT/μg to 1,000 pmol CT/μg total protein.

### Determination intracellular CT and CycCT levels – LC/MS/MS method

100 µL of the deproteinized cell supernatant was mixed with 10 µL of the internal standard and 500 µL methanol (Fisher Scientific, Ottawa, ON). Samples were vortexed and spun down at 13,000 rpm for 5 min. The supernatant was transferred into a glass test tube and loaded onto the Microvap (Organomation, Berlin, MA) at 37°C to evaporate the excess solvent. Dry residue was dissolved in 100 µL 3M butanol·HCL (Regis, Morton Grove, IL) by vortexing and incubated at 60 °C for 30 min. After cooling to room temperature, derivatized samples were transferred onto the Microvap at 37 °C to evaporate the excess of solvent. Dry residue was resuspended in 700 µL methanol and transferred into a 2 mL glass vial.

CT and CycCT were analyzed on ExionLC AD UHPLC system coupled with QTRAP 6500plus (AB Sciex LLC, Framingham, MA). The metabolites separation was performed using gradient binary elution at a flow rate of 0.7 mL/min and a temperature at 45 °C on a Kinetex C18 100 Å, 5 µm, 100 × 4.6 mm LC column (Phenomenex Inc., Torrance, CA). Solvent A consisted of 0.5 mmol/l ammonium formate, 0.1% (v/v) formic acid in water and solvent B consisted of 0.5 mmol/l ammonium formate, 0.1% (v/v) formic acid in methanol. The mobile phase was used at 100% A at 0 min; 100% B at 5.0 min; 100% B at 7.5 min; 100% A at 7.55 min; 100% A at 10 min. The injection volume was 1 µL. The mass spectrometry was performed at the positive ionization and multiple reaction monitoring scan mode. The metabolites ion transitions were as follows: CT—188.2 → 146.3, CycCT—200.2 → 144.2, CT-d3—191.2 → 149.1. The ion source parameters were set at TEM – 600 °C, de-clustering potential—60.0, capillary voltage—5500 V, curtain gas—30, GS1—30, and GS2—20. Data processing and quantification of metabolites was performed with Analyst 1.7.0 software (AB Sciex LLC, Framingham, MA).

External calibrators for CT (Sigma-Aldrich Canada Co., Oakville, ON) and CycCT (TRC, Toronto, ON) were prepared as a mixture of both compounds at final concentrations of 500, 200, 100, 50, 20, 10, 5, 2.5 and 0 µM. The CT-d3 (Sigma-Aldrich Canada Co., Oakville, ON) internal standard was used at concentration of 100 µM. Calibrators and the internal standard were stored at − 20 °C until use. The linear range in protein-containing matrix is approximately 10 μM to 5 mM for CT and 40 μM to 2 mM for CycCT.

### Western blotting

Cells from one 10 cm plate were washed with PBS, scraped, and pelleted using low speed centrifugation. Cell pellet was resuspended in 300 µL of lysis buffer (1% Triton-100, 50 mM Tris pH 7.4, 150 mM NaCl) by vortexing and spun down at 13,000 rpm for 1 min to separate insoluble cellular debris. After measuring cell lysates protein concentration by BCA Protein Assay (Thermo Scientific), 50 µg of total protein were denatured in 1 × Laemli buffer for 15 min at 56 °C and separated by 4–15% SDS-PAGE (Bio-Rad). Protein gels were transferred to the polyvinylidene fluoride (PVDF) membrane (Bio-Rad) in transfer buffer (10 mM CAPS pH 11, 10% methanol) for 1 h at 4 °C, blocked with 5% skim milk in TBST for 1 h at room temperature, followed by the primary antibodies for 1.5 h at room temperature and the secondary antibodies for 1 h at room temperature. The primary antibodies used in Western blot analysis were rabbit AGAT antibody (kindly provided by Dr. Theo Walliman) and commercial rabbit anti human AGAT polyclonal Ab raised to the N-terminus of AGAT (Novus Biologicals, LLC Cat. No. NBP1-89,211) at 1:500 dilutions, and rabbit polyclonal GAPDH antibody (Sigma) at 1:10,000 dilutions. The secondary antibodies were horseradish peroxidase conjugated donkey anti-rabbit (Jackson Immunology) at 1:10,000 dilutions. Proteins were detected using Clarity Western ECL Substrate (Bio-Rad, USA) and visualized using the Bio-Rad ChemiDoc MP imaging system.

### Luciferase assay

HAP1 cells expressing AGAT tagged at the C-terminus with NLuc were grown for two days in Iscove’s media supplemented with 10% dialysed FBS. Approximately, 25,000 cells (in 100 µL) were aliquoted into each of the wells of tissue culture treated opaque 96 well plate. Two dilution series were prepared one set consisting of a 24-step serial 1.25 dilution starting at 100mM and another one starting at 10 mM (both in Iscove’s media containing 10% dialysed FBS). The diluent in all cases was Iscove’s media with 10% dialysed FBS. Cells were treated with an equal volume of CT dilution series in triplicate overnight (24h). Following treatment, media was removed and re-plated with 100 µL Iscove’s media containing 10% FBS and 10 µM Coelenterazine (1/100 dilution of 1mM Coelenterazine H stock in acidified ethanol). Luminescence readings were performed on a Neo2 Multimodal HTS plate reader using the Lum 485 optical filter cube.

### Statistical analysis

Experiments to determine intracellular CT or fraction remaining luciferase activity represent biological replicates performed in triplicate or quadruplicate, as noted in the figure legends. Prism GraphPad 9 (GraphPad Software, Boston, US) was used to fit data to different models and prepare all graphs. Error bars are shown as standard deviation in all graphs. To extract IC50 values, data presented in Figs. 4C and 7D were modeled using Y = Bottom + (Top–Bottom)/(1 + 10^(X-LogIC50)) where Y represents the fraction remaining luciferase activity and X corresponds to the log of the intracellular CT. Km and Vmax values associated statistics were determined following modelling either intracellular CT or Fraction remaining luciferase activity to the model Y = Vmax*X/(Km + X) and were performed within GraphPad Prism 9. Two phase decay curves in Figures. 1F, 2, 4A,B, 6, 7A,B,C were fit to the model SpanFast = (Y0-Plateau)*PercentFast*0.01; SpanSlow = (Y0-Plateau)*(100-PercentFast)*0.01; Y = Plateau + SpanFast*exp(-KFast*X) + SpanSlow*exp(-KSlow*X, where X is the CT concentration and Y represents the fraction remaining luciferase activity or intracellular CT concentration.

### Supplementary Information

Supplementary Information.

## Data Availability

The datasets generated during and/or analysed during the current study are available in the GenBank repository. HAP1_WT_AGAT_NLUC_C8.32.4 GenBank OR492632; CRTKO_AGAT_NLUC_s5.27.6 GenBank OR492633; AGAT_NLUC_donor GenBank OR492634, as supplemental file, and on reasonable request from the communicating author andreas.schulze@sickkids.ca.

## Abbreviations

AGAT-NLuc: Endogenous AGAT gene tagged at the c-terminus (coded by exon 9) with Nluc
CrT: Creatine transporter *SLC6A8*
CrT^WT^: HAP1 wild type cell line expressing the creatine transporter
CrT^KO^: HAP1 creatine transporter knockout line
CrT-GFP: Overexpressed CrT with in frame C-terminal GFP. Expression from stably integrated CMV CrT GFP plasmid construct
CT: Creatine
CycCT: Cyclocreatine
FBS: Fetal bovine serum
HDR: Homology directed repair
IC50: Half-maximal inhibition constant
Km: Half-maximal uptake constant
NLuc: Nanoluc luciferase

## Cell lines discussed

(HAP1) WT alias CrT^WT^: Parental cell line
(HAP1) AGAT-NLuc alias CrT^WT^ AGAT-NLuc: HAP1 containing one copy of AGAT-NLuc gene
(HAP1) CrT^KO^ AGAT-NLuc: HAP1 homozygous CrT knockout containing one copy AGAT-NLuc gene
(HAP1) CrT^KO^: HAP1 homozygous for CrT knockout
(HAP1) CrT^KO^ AGAT-NLuc CrT-GFP: HAP1 homozygous CrT knockout containing one copy *AGAT-NLuc* gene stably expressing CrT c-terminal GFP fusion under control of CMV promoter
