# Supplementary material for: Evidence of an intracellular creatine-sensing mechanism that modulates creatine biosynthesis via AGAT expression in human HAP1 cells
Source: Sci Rep. 2023 Dec 16;13:22392. doi: 10.1038/s41598-023-49860-1 (PMC10725494; doi:10.1038/s41598-023-49860-1)
Supplement: Supplementary file 1 — Supplementary Information. [file 41598_2023_49860_MOESM1_ESM.pdf]

Supplemental information

Table 1S – Primers for construction AGAT Ex9 grna, GATMex9nluc Donor, primers to detect insertion of donor and expression of AGAT nluc fusion mRNA

PRIMER ID	PRIMER SEQUENCE ('5->3')	PURPOSE
**GRHUGATM-EX9FOR**	CACCGCAGTCCTACTTGGACTGAAC	gRNA targeting ex9 GATM
**GRHUGATM-EX9REV**	AAACGTTCAGTCCAAGTAGGACTGC	gRNA targeting ex9 GATM
**AGATEX9LEFTFORASCI**	CCTAGGCGCGCCCTCTGTTGACTTTTTGGAGAAG	Left donor arm
**AGATLEFTDONOR REVV2**	GGAGTGAAGACCTACGCTGCCCCCTCCGCCGTCCAAGTAGGACTGTAAG	Left donor arm
**NLUC_AGAT3'UTR-REV**	ccacaagCTCCATCAGGCCTGTTCATTAGACGTTGATGCGAGCTGAAGC	Nluc insertion
**AGAT_CT_NLUC-FOR**	TACTTGGACGGCGGAGGGGGCAGCGTGTTCACACTCGAAGATTTC	Nluc insertion
**AGATEX9RIGHTREVPACI**	GGAGTTAATTAAGAGACAATTACCTGGTTCTACTTAGG	Right donor arm
**AGATRIGHTDONORFWD**	TGGAGTGAAGACGTAATGAACAGGCCTGATGGAG	Right donor arm
**QPCREX9GATMREV2**	ggcttacgacccctgttaaggag	Detect AGAT-nluc fusion mRNA
**QPCREX9GATM REV**	agaatgaaccttgcccctaagc	Detect AGAT-nluc fusion mRNA
**QPCREX9GATMFOR**	gtatcactaccattaaagttaac	Detect AGAT-nluc fusion mRNA
**QPCREX8GATMFOR**	gttatggtggatgccaatgaag	Detect AGAT-nluc fusion mRNA
**GATM-GFP-INS1-FOR**	atctgctcagtctattgacttgtgaatag	Detect insertion donor in ex9
**GATM-GFP-INS1-REV**	gagacaattacctggttctacttagg	Detect insertion donor in ex9

igure

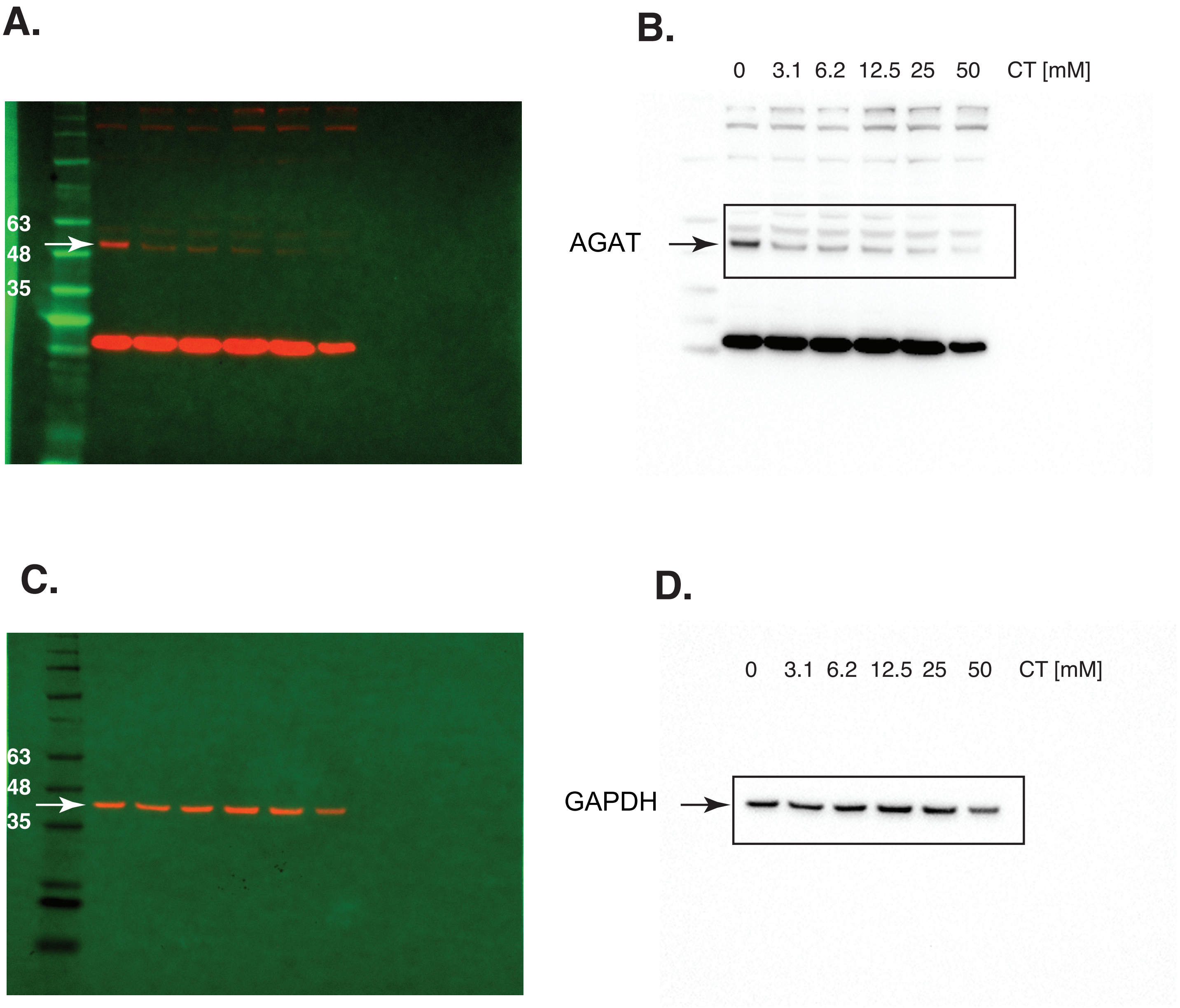

Figure S1. Uncropped AGAT and GAPDH western blots shown in Figure 1A. A. C., Composite image of merged 647 nm fluorescent channel (colored red) showing the prestained molecular weight standards and high sensitivity chemiluminescent channel (colored green) showing the AGAT (A) and re-probed GAPDH (C) western blot following addition of the chemiluminescent substrate. White arrows point to the relevant bands. Molecular weights shown in kDa. B.D. Gray scale image of the chemiluminescent channels in A and C. Boxed region corresponds to the cropped image shown in Fig. 1A AGAT (B) and GAPDH (D).

igure

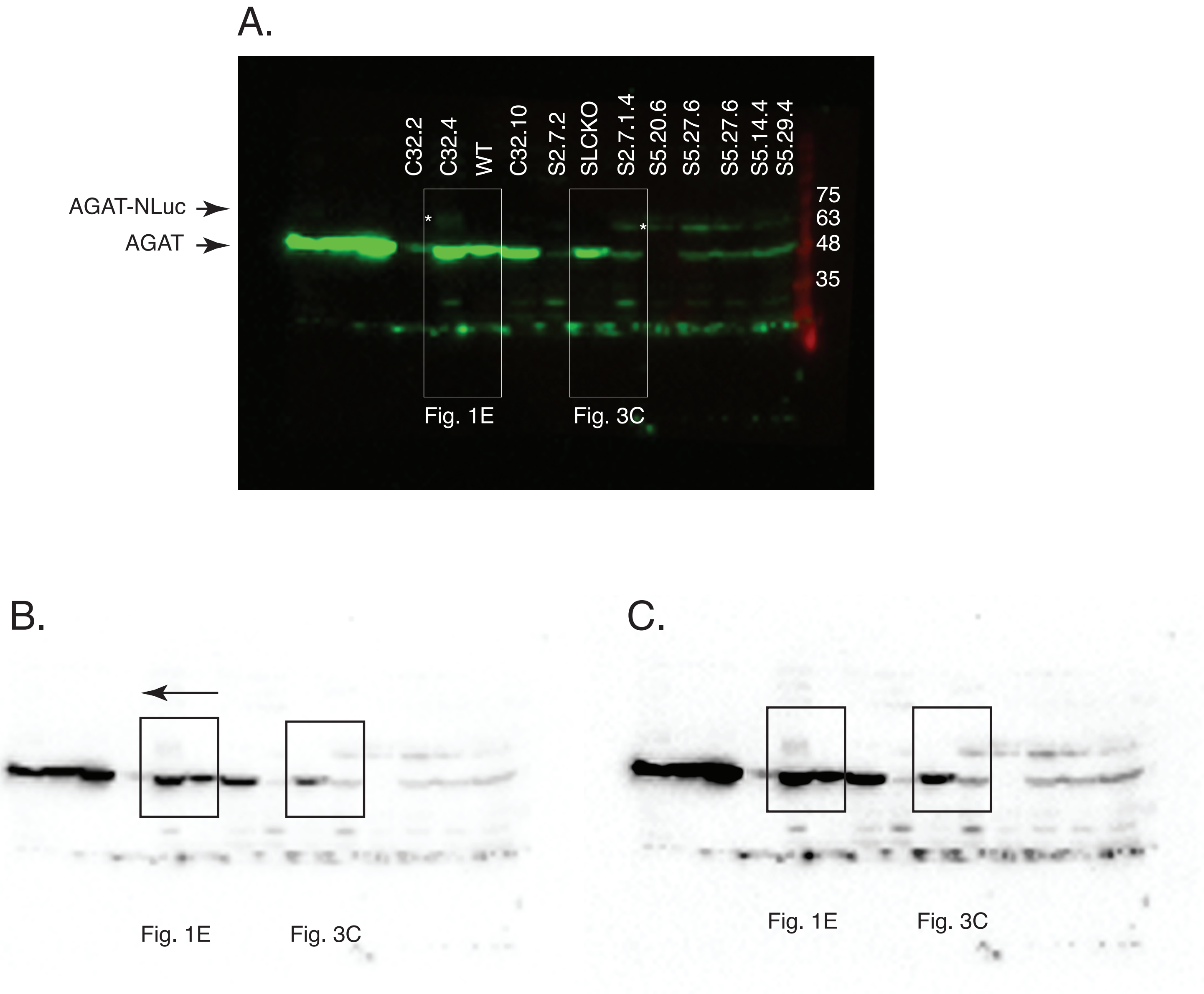

Figure S2. A. Uncropped western blots used to generate panels in Fig. 1E and Fig. 3C. Lanes used in these figures are boxed. Topmost panel is a composite image of merged 647 nm fluorescent channel (colored red) showing the pre-stained molecular weight standards and high sensitivity chemiluminescent channel (colored green) showing the western blot following addition of the chemiluminescent substrate. B. Reverse grayscale image of the chemiluminescent channel shown in A. Boxed regions correspond to the cropped images used in Figs. 1E and 3C. As indicated by the arrow, cropped image in Fig. 1E is rotated 180 degrees relative that in the uncropped image. C. The brightness of the bands in A. has been increased to better highlight the band corresponding to the AGAT-NLuc fusion protein.

Figure S3

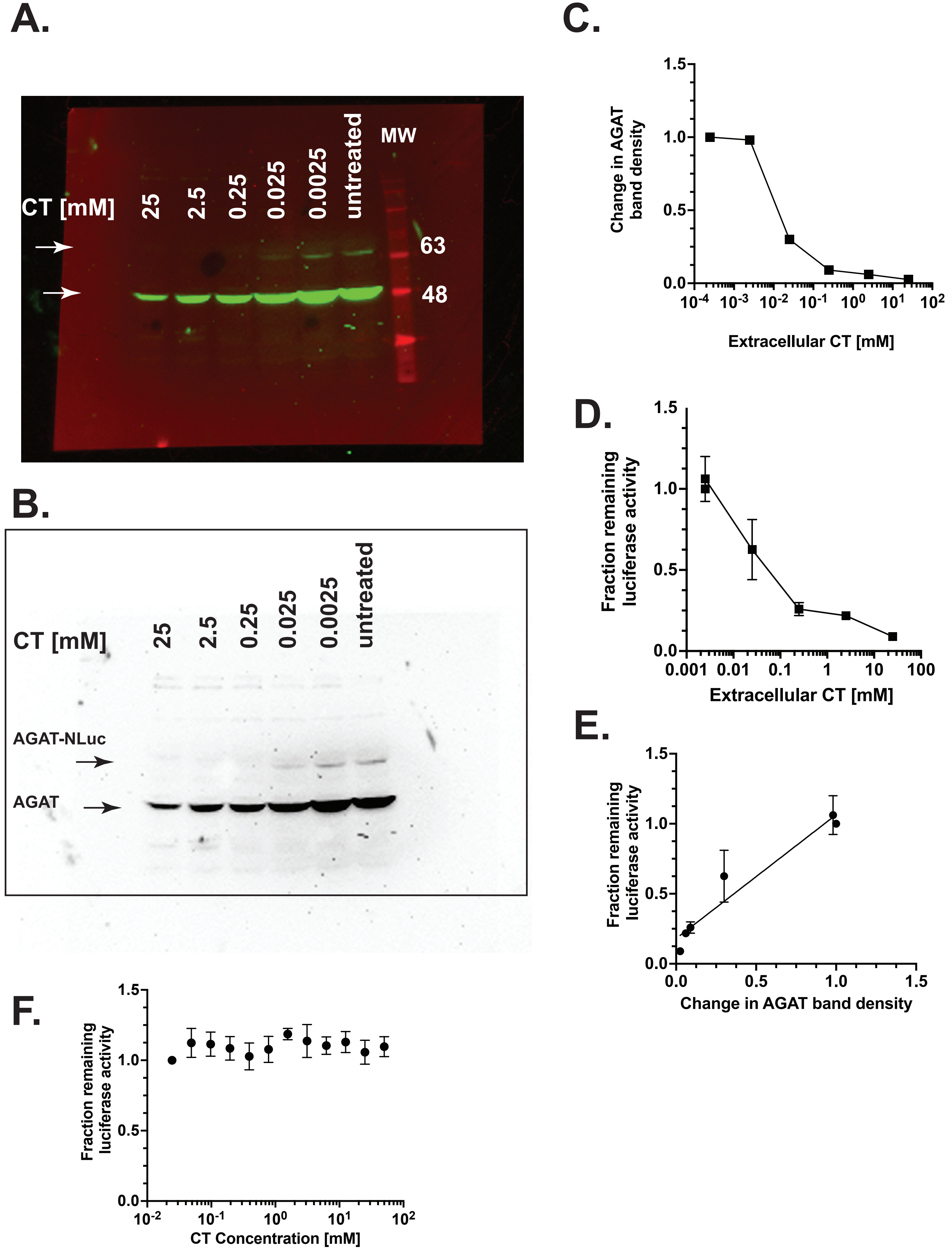
A.
CT [mM]
25
2.5
0.25
0.025
0.0025
untreated
MW
63
48
B.
CT [mM]
25
2.5
0.25
0.025
0.0025
untreated
AGAT-NLuc
AGAT
C.
Change in AGAT band density
Extracellular CT [mM]
D.
Fraction remaining luciferase activity
Extracellular CT [mM]
E.
Fraction remaining luciferase activity
Change in AGAT band density
F.
Fraction remaining luciferase activity
CT Concentration [mM]

Figure S3 (cont.)

G.

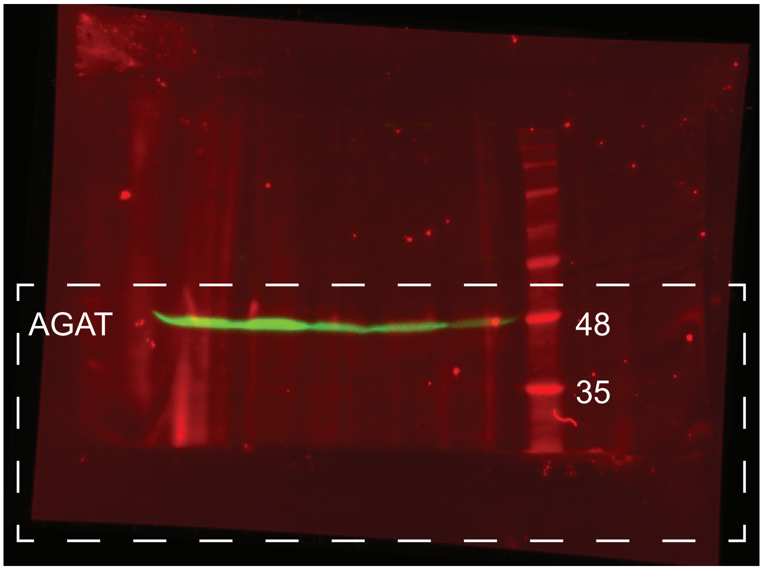

H.

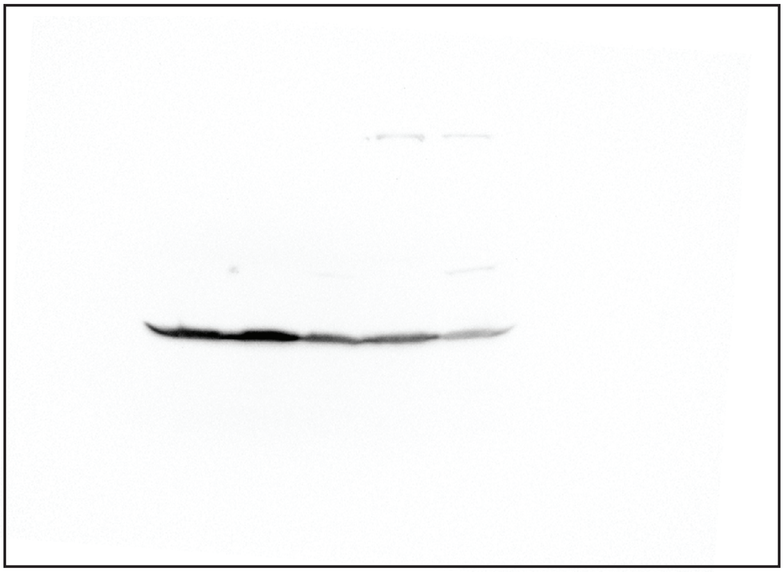

I.

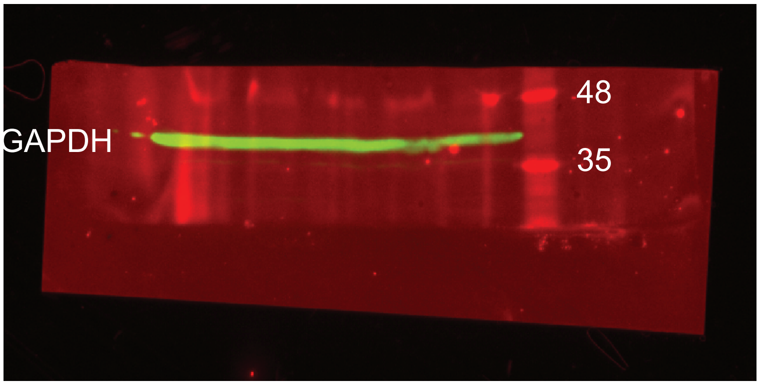

J.

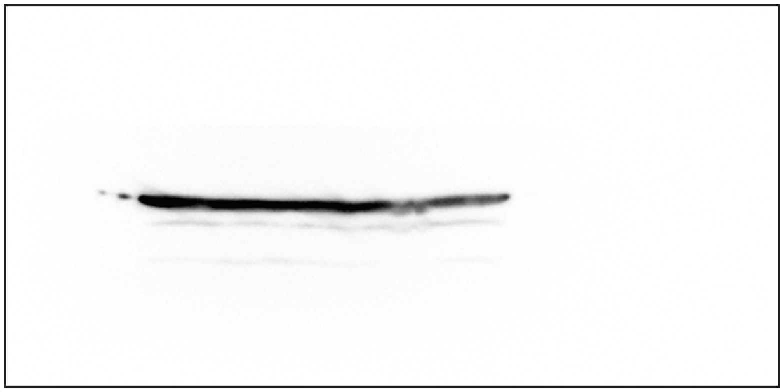

Figure S3. Change in AGAT-NLuc activity parallels decrease in endogenous AGAT protein levels with increasing dose of CT. CrT^{KO} AGAT-NLuc cells grown in dialyzed media for one day prior to 24 hr treatment with an escalating dose of CT added into dialyzed media. **A.** Composite image of merged 647 nm fluorescent channel (colored red) showing the pre-stained molecular weight (MW) standards and high sensitivity chemiluminescent channel (colored green) showing the AGAT. Arrows point to endogenous AGAT band and AGAT-NLuc fusion protein.
B. Inverted gray scale image chemiluminescent channel shown in A. **C.** Change in endogenous AGAT band intensity (quantified within ImageLab Biorad gel imaging software) with increasing dose of extracellular CT. **D.** Change in AGAT-NLuc luciferase activity in the CT treated CrT^{KO}AGAT-NLuc lysate used for the western blot. **E.** Change in endogenous AGAT band intensity following CT treatment plotted versus change in AGAT-NLuc luciferase activity following CT treatment. **F.** AGAT-NLuc luciferase activity is not directly inhibited by CT. Cells (10,000,000) from HAP1 CrT^{WT}AGAT-NLuc (C32) clone were sonicated in 1 mL buffer containing 50 mM Tris 7.5, 50 mM NaCl. Aliquots (10 µL) of lysate (n=3) were treated with an escalating dose of CT (150 µL). Following addition of an equal volume (150 µL) of coelenterazine (20 µM) Luminescence was quantified on a Varioskan LUX Multimode Microplate Reader (Thermo Fisher Scientific, USA). Plot shows change in luciferase activity normalized to luciferase activity in buffer without added CT.
G. Uncropped Fig. 2E AGAT - false color chemiluminescent (green) and 647 nm fluorescent (red) channel. Bottom portion of blot cut and reprobed with GAPDH (white box). **H.** Reverse gray scale image of chemiluminescent channel in left panel.
I. The bottom portion of AGAT blot was cut (white dashed box above) and reprobed with GAPDH Ab. AGAT Ab from Novus Biologicals LLC, USA (Cat. No. NBP1-89211, Rabbit Anti Human AGAT N-terminus) used in panels A, B, G, and I. Uncropped Fig. 3E GAPDH - false color chemiluminescent (green) and 647 nm fluorescent (red) channel. **J.** Reverse gray scale image of chemiluminescent channel in left panel.

Figure S4

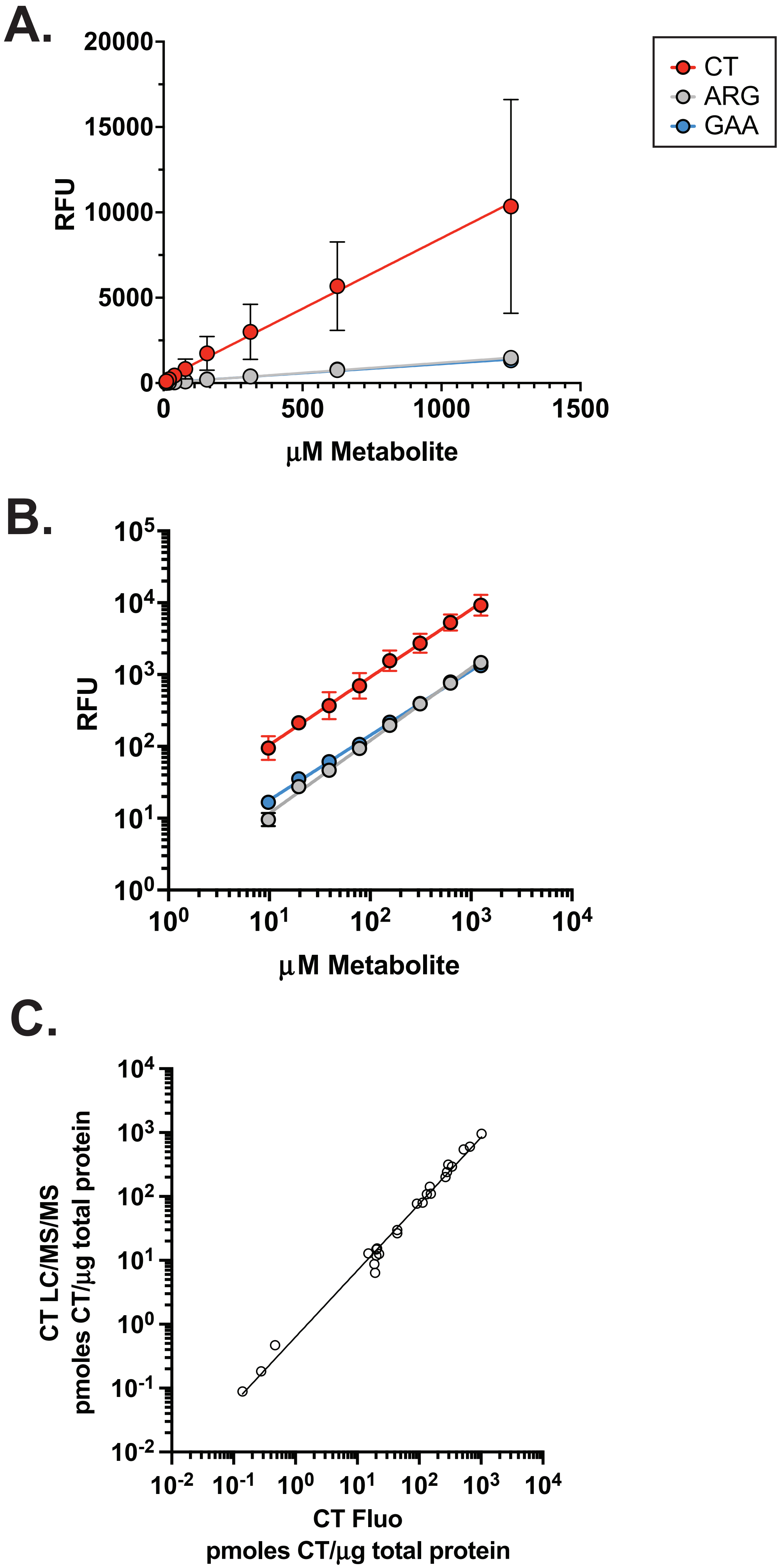
A.
RFU
20000
15000
10000
5000
0
0
500
1000
1500
μM Metabolite
CT
ARG
GAA
B.
RFU
10^5
10^4
10^3
10^2
10^1
10^0
μM Metabolite
C.
CT LC/MS/MS
pmoles CT/μg total protein
10^4
10^3
10^2
10^1
10^0
10^{-1}
10^{-2}
CT Fluo
pmoles CT/μg total protein

Figure S4. When CT reacts with ninhydrin it produces a more fluorescent product than either arginine or guanidinoacetate at the same concentration. **A.** Different concentrations of the three metabolites CT, Arg or GAA were reacted with ninhydrin under alkaline conditions. Fluorescence of the product was determined and plotted versus test concentration of the selected metabolites (n=3, mean ± SD). **B.** Same plot as in A, except Y-axis uses a logarithmic scale. **C.** Comparison of intracellular CT values determined using LC/MS/MS versus ninhydrin. Lysates from HAP1 cells were treated with an escalating dose of CT and intracellular CT concentration was determined using either LC/MS/MS or using the ninhydrin fluorescence assay.
